# The impact of a 2-year Etanercept administration on growth of patients with juvenile idiopathic arthritis

**DOI:** 10.1186/1546-0096-9-S1-P149

**Published:** 2011-09-14

**Authors:** M Trachana, P Triantafyllou, A Vraka, E Kaitalidou, P Pratsidou-Gertsi

**Affiliations:** 1 1st Department of Pediatrics, Aristotle University, Ippokration General Hospital, Thessaloniki, Greece

## Background

Published data from various regions evidenced that the administration of Etanercept (ET) restored auxological retardation attributed to the process of the chronic joint inflammation.

## Aim

To assess the impact of ET on growth pattern in Greek JIA patients who had a resistant poly- or an oligoarthritis. Methods: Data of 24 JIA patients (F:M 19:5, 20/24 with a polyarthritis) who had received ET at a median age of 6.7 yrs (1.9-14.5 yrs) escorted by their auxological parameters were collected. Standard Deviation Scores for Height (HSDS) and Body Mass Index (BMISDS) were calculated according to sex- and age- matched percentiles of the healthy Greek population, 2 and 1 yrs pre-ET treatment, and 1 and 2 yrs thereafter. Growth velocity was defined as the change in Height SDS (ΔHSDS) and in BMI (ΔBMISDS) and compared to the time of ET initiation (baseline).

## Results

At baseline 16/24 pts had a growth retardation (median ΔHSDS –0.56); 11/16 showed a significant increase 1yr post-treatment (ΔHSDS +0.29, p<0.001) which did not further increase (2nd yr: ΔHSDS +0.15). Similarly, at baseline, BMI was retarded in 14/24 pts (median ΔBMISDS-0.6); in 10/14, BMI increased 1 and 2 yrs post-ET (ΔBMISDS +0.31, +0.12 respectively, p<0.001) and remained stable thereafter. Growth and BMI improvements were not associated either with age of ET initiation, or gender, or presence of ANA, or disease duration, or JIA course or MDVAS.

## Conclusion

ET treatment can restore the linear growth and BMI of JIA patients irrespectively of their demographic or clinical profile.

